# Modulation of TRPV4 protects against degeneration induced by sustained loading and promotes matrix synthesis in the intervertebral disc

**DOI:** 10.1096/fj.202201388R

**Published:** 2023-02

**Authors:** Garrett W. D. Easson, Alireza Savadipour, Akila Anandarajah, Leanne E. Iannucci, Spencer P. Lake, Farshid Guilak, Simon Y. Tang

**Affiliations:** 1 Department of Mechanical Engineering, Washington University in St. Louis, St. Louis, Missouri, USA; 2 Department of Orthopaedic Surgery, Washington University in St. Louis, St. Louis, Missouri, USA; 3 Shriner’s Hospital for Children – St. Louis, St. Louis, Missouri, USA; 4 Department of Biomedical Engineering, Washington University in St. Louis, St. Louis, Missouri, USA

**Keywords:** annulus fibrosus, extracellular matrix, inflammation, intervertebral disc, NF-kappa B, TRPV cation channels, vascular endothelial growth factor A

## Abstract

While it is well known that mechanical signals can either promote or disrupt intervertebral disc (IVD) homeostasis, the molecular mechanisms for transducing mechanical stimuli are not fully understood. The transient receptor potential vanilloid 4 (TRPV4) ion channel activated in isolated IVD cells initiates extracellular matrix (ECM) gene expression, while TRPV4 ablation reduces cytokine production in response to circumferential stretching. However, the role of TRPV4 on ECM maintenance during tissue-level mechanical loading remains unknown. Using an organ culture model, we modulated TRPV4 function over both short-(hours) and long-term (days) and evaluated the IVDs’ response. Activating TRPV4 with the agonist GSK101 resulted in a Ca^2+^ flux propagating across the cells within the IVD. Nuclear factor (NF)-κB signaling in the IVD peaked at 6 h following TRPV4 activation that subsequently resulted in higher interleukin (IL)-6 production at 7 days. These cellular responses were concomitant with the accumulation of glycosaminoglycans and increased hydration in the nucleus pulposus that culminated in higher stiffness of the IVD. Sustained compressive loading of the IVD resulted in elevated NF-κB activity, IL-6 and vascular endothelial growth factor A (VEGFA) production, and degenerative changes to the ECM. TRPV4 inhibition using GSK205 during loading mitigated the changes in inflammatory cytokines, protected against IVD degeneration, but could not prevent ECM disorganization due to mechanical damage in the annulus fibrosus. These results indicate TRPV4 plays an important role in both short- and long-term adaptations of the IVD to mechanical loading. The modulation of TRPV4 may be a possible therapeutic for preventing load-induced IVD degeneration.

## 1 | INTRODUCTION

Low back pain (LBP) is one of the most prevalent musculoskeletal diseases in the U.S., and it is the leading cause of disability.^1–3^ The degeneration of the intervertebral disc (IVD), the soft cartilaginous tissue between the vertebrae, is a significant contributor to low back pain.^4–6^ The IVD experiences continuous mechanical loading that enable motion and load transmission in the skeleton. While mechanical stimulation is crucial for IVD homeostasis and maintenance, over-loading leads to tissue damage with the subsequent expression of inflammatory cytokines such as interleukin (IL)1β, IL-6, and IL-8 in IVD cells.^7–12^ Although the transient expression of inflammatory cytokines such as IL-6 and vascular endothelial growth factor A (VEGFA) has an essential regulatory role in tissue regeneration, fracture repair, and wound healing,^13–15^ their sustained expression can eventually cause the release of catabolic enzymes, initiate immune cell recruitment, and provoke vascular and neurite invasion of the IVD.^8,16–19^

Mechanosensitive ion channels are activated by mechanical stimuli and allow for the transport of ions across the cell membrane, and they are hypothesized to participate in the IVD’s responsiveness to its loading environment.^20,21^ One candidate mechanosensitive channel in the IVD is transient receptor potential vanilloid 4 (TRPV4), a nonselective cation channel in the TRP family of channels.^22,23^ TRPV4 is an important osmo- and mechanoregulator in a variety of cell types, including chondrocytes, osteocytes, cardiovascular endothelial cells, and renal epithelial cells.^24–26^ TRPV4 is crucial for mechanosensing in articular cartilage and has been shown to regulate the anabolic response of chondrocytes to mechanical loading.^27–29^ Similar to articular cartilage in both its motion-enabling joint function and matrix constituents, the mechanosensing mechanisms may be conserved in the IVD. Moreover, the application of load to both cartilage and the IVD causes perturbations in the local osmotic environment that triggers a Ca^2+^ signaling response.^30,31^ However, it is unknown whether this response is mediated by TRPV4.

In IVD cell culture, inhibiting TRPV4 reduces the hypo-osmotic-mediated production of IL-1β and IL-6, and the production of IL-6 and IL-8 mediated by high-magnitude strains.^32,33^ Conversely, activation of TRPV4 in IVD cells promotes the expression of matrix protein genes including ACAN and COL2A1.^34^ We, therefore, hypothesized that TRPV4 regulates the mechanotransduction of physiologically relevant loading in the IVD. To investigate the complex interactions between physiologic loading, maintenance of the extracellular matrix (ECM), and modulation of TRPV4, we used an organ culture IVD system that allowed us to apply compression in a sustained manner, in the presence or absence of a TRPV4 agonist or antagonist, and observe outcomes on the whole IVD.

We first examined the effects of transient TRPV4 activation using a small molecule agonist (GSK1016790A, i.e., GSK101) on the cellular level Ca^2+^ across the IVD. We then compared the IVDs’ adaptations resulting from prolonged activation of TRPV4 and corresponding hypo-osmotic conditions. Finally, we evaluated the role of TRPV4 under sustained compression of the whole IVD at physiologically relevant loads. Our results show that the transient, single-bout activation of TRPV4 in IVD organ culture increases the intracellular Ca^2+^ flux and a cascade of NF-κB signaling and production of IL-6. The repeated activation of TRPV4 over a one-week period improved the compressive stiffness of the IVD and increased glycosaminoglycan (GAG) content and hydration in the nucleus pulposus (NP). Further, the inhibition of TRPV4 during the repetitive hypo-osmotic conditions blunted IL-6 production by the IVD. The sustained mechanical compression of IVDs resulted in degeneration along with prolonged expression of IL-6 and VEGFA. The inhibition of TRPV4 during this sustained loading alleviated the degeneration and protected from the increases of IL-6 and VEGFA, but it did not prevent the load-induced collagen disorganization in the annulus fibrosus. Taken together, the transient activation of TRPV4 initiates the cascade of Ca^2+^ signaling, NF-κB signaling, and IL-6 expression, with the repeated activation of TRPV4 increasing GAG accumulation and the stiffness of the IVD ECM. Inhibition of TRPV4 mitigated the degenerative effects of sustained mechanical loading. These results suggest that modulation of TRPV4 could provide a novel approach for preventing degeneration of the IVD following deleterious loading.

## 2 | MATERIALS AND METHODS

### 2.1 | Animals

**FIGURE 1:**
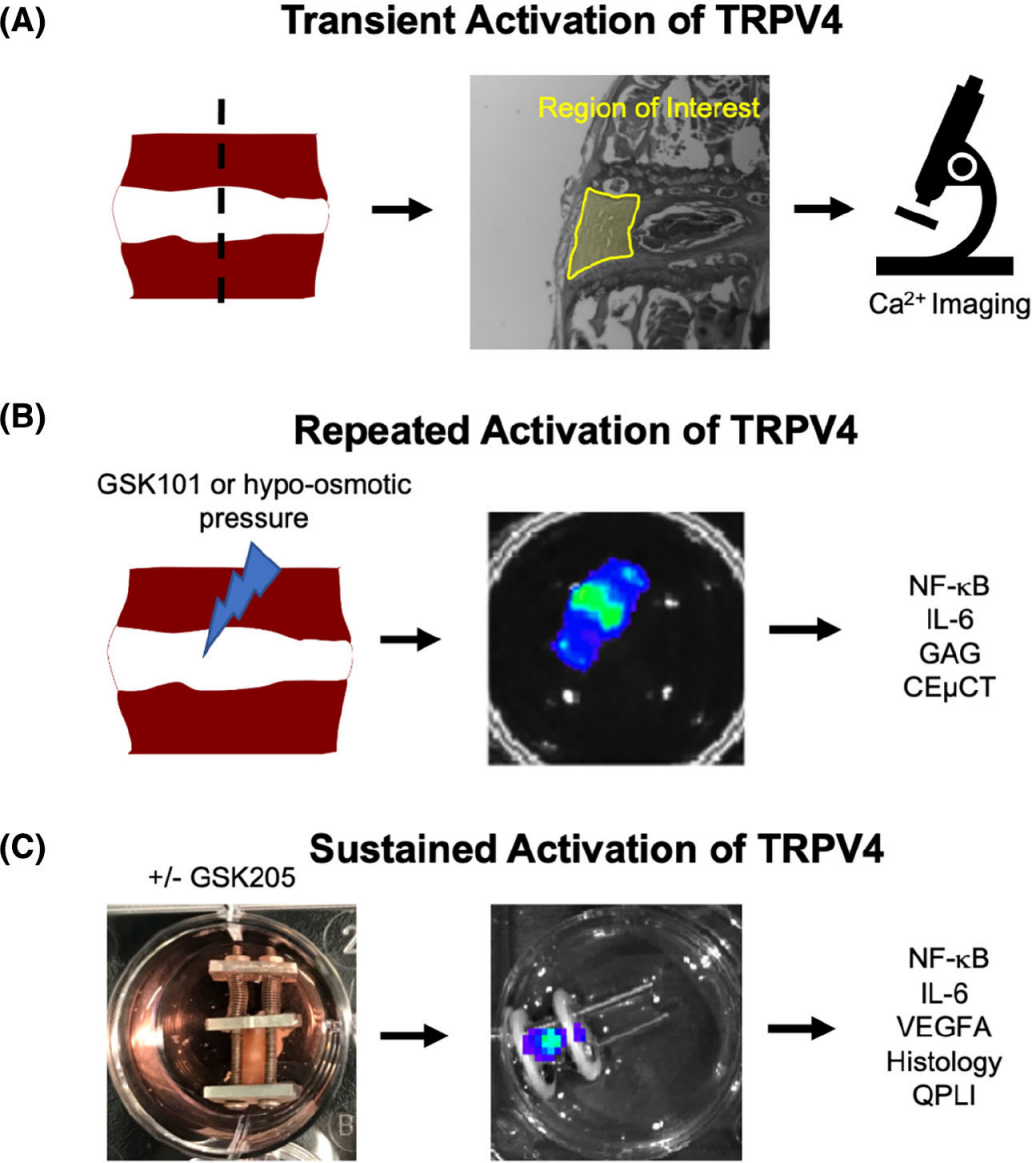
Functional Spine Units including the IVD were extracted from 16-week-old mice carrying the NF-κB -luciferase reporter transgene, and then were divided into 3 sets of organ culture experiments: (A) Observation of calcium flux following a single, transient bout of TRPV4 activation. Fluorescent Ca^2+^ signals were observed using confocal microscopy immediately following GSK101 administration (*n* = 3); (B) the repeated activation of TRPV4 using GSK101 (*n* = 15) or by hypo-osmotic pressure (*n* = 8); and (C) the sustained activation through static compression throughout the culture period (*n* = 11). The outcome measures utilized in (B) and (C) include NF-κB-luciferase activity, IL-6 and VEGFA cytokine production measured by ELISA, glycosaminoglycan (GAG) composition measured by the dimethylmethylene blue (DMMB) assay, IVD structure measured by contrast-enhanced μCT (CEμCT), IVD degeneration measured by histologic grading, and IVD tissue organization measured by quantitative polarized light imaging (QLPI).

All animal experiments were performed in compliance with the Washington University in St. Louis Institutional Animal Care and Use Committee. Sixteen-week-old mice carrying the NF-κB -luciferase reporter transgene (FVB. Cg-Tg(HIV-EGFP,luc)8Tb/J; JAX) were used. The mice were euthanized with CO_2_ for 2 min and then disinfected in 70% ethanol. The whole lumbar spine was then removed and separated into three functional spine units (FSUs): L1/L2, L3/L4, and L5/L6. All excess tissue including the spinal cord was removed. FSUs were immediately placed in culture media in 24-well plates and pre-conditioned for 7 days prior to all experiments. Culture media consisted of 1:1 Dulbecco’s modified Eagle’s medium: Nutrient mixture F-12 (DMEM:F12) supplemented with 20% fetal bovine serum and 1% penicillin–streptomycin.^35^ The FSUs were cultured under the following conditions (37°C, 5% CO_2_, 100% humidity) and were assigned to one of three distinct experiments: transient activation of TRPV4 repeated activation of TRPV4 and sustained activation of TRPV4 (Figure 1). Our prior work demonstrated that these culture conditions maintain the viability of all cells in the IVD, including those in the cartilaginous end-plate, but not cells in the bone marrow space or osteocytes.^35^

### 2.2 | Confirmation of TRPV4 function through Ca^2+^ signaling

TRPV4 is a preferential Ca^2+^ ion channel, and its activation results in fluxes of intracellular Ca^2+^. To confirm that TRPV4 is present and functional in our functional spine unit (FSU) cultures (*n* = 3), we measured changes in intracellular Ca^2+^ concentrations using ratio imaging of Fluo-4 and Fura Red Ca^2+^ indicators. The FSUs were incubated with Fluo-4 and Fura Red Ca^2+^ indicators for 1 h. Changes in intracellular Ca^2+^ were observed in real-time (Zeiss LSM 880) and the ratio of Fura Red intensity (decreases with low Ca^2+^) to Fluo-4 intensity (increases with high Ca^2+^) were computed. The Ca^2+^ gradient was quantified using a custom MATLAB script by dividing the green channel intensity by the red channel intensity frame-by-frame to obtain relative fluorescent units (RFU). The fluorescence was observed using confocal microscopy at ~150 μm of depth into the annulus fibrosus (AF) to evaluate the activation of TRPV4 in AF cells in their native extracellular matrix. DMSO was added to the IVD at the start of the live imaging session, and then cells were monitored for approximately 200 s. Following this period, 1 μM GSK101 was added to the culture to agonize TRPV4. Concentrations of 100 μM and 1 mM of GSK101 were subsequently added to the culture media of respective IVDs with each IVD receiving just one concentration to determine the dose-dependent response.

### 2.3 | Repeated TRPV4 activation using the GSK101 agonist in FSU culture

FSUs in culture were either incubated with DMSO (*n* = 12) or the TRPV4 agonist GSK101 (2 μM; *n* = 14) for seven consecutive days for three hours each day. Continuously high intracellular Ca^2+^ concentrations are toxic for cells, and sustained Ca^2+^ saturation can induce apoptosis. Thus, we selected a dose of GSK101 that activates but does not saturate intracellular Ca^2+^.^36^ All treatment was delivered in 1 ml of fresh culture media containing either DMSO or GSK101. After 3 h, the IVDs were returned to fresh media under the prescribed conditions.

### 2.4 | Real-time observation of NF-κB signaling

NF-κB signaling was observed longitudinally for several experiments. Luciferin (30 mg/ml) was added to the culture media, which then binds to the luciferase transgene to produce detectable bioluminescence quantifying the degree of NF-κB signaling in the IVD. Bioluminescence was measured 30 min following luciferin addition at either: Time 0 h, 3 h, 6 h, 9 h, or 24 h for the short-term study; or Days 0, 3, 7, and 14 of the organ culture for the long-term study. Samples were imaged using an IVIS Imaging System (Xenogen Corp.) at a 10 s exposure time. Lipopolysaccharide (LPS) was used to produce a positive NF-κB control (1 μg/ml, Sigma Aldrich: L8274). The Day 0 time point is the baseline prior to the application of experimental conditions. Following every imaging timepoint, the FSUs were transferred to fresh media.

### 2.5 | Cytokine production by measured ELISA

Media was collected from the FSU cultures on Days 0, 3, 7, and 14 of the primary study and stored at −80°C. The concentrations of IL-6 and VEGFA were determined using an enzyme-linked immunosorbent assay (Mouse IL-6 ELISA kit, ThermoFisher; Mouse VEGF-A DuoSet ELISA kit, R&D Systems). All media was collected 2 days following media change, such that measured cytokine concentration reflected 2 days of production.

### 2.6 | Contrast-enhanced micro-computed tomography

To evaluate structural changes, IVDs were imaged using contrast-enhanced micro-computed tomography (CEμCT) with ioversol as the contrast agent.^37^ Intact IVDs were incubated in 50% Ioversol (OptiRay 350, Guerbet Pharma) at 37°C for 8 h prior to imaging. Following incubation, samples were scanned using Scanco40 microCT system (Zurich, Switzerland) at 45 keVp, 177 μA, 10.5 μm voxel size, and 300 ms integration.

### 2.7 | Mechanical behavior of the whole IVD

The mechanical behavior of the IVDs was quantified using displacement-control dynamic compression testing (BioDent, Active Life Scientific).^38^ After FSUs were adhered to aluminum platens, disc height was determined by calipers and used to determine the input strain values. Samples were placed in a phosphate-buffered saline bath and preloaded to 0.02 N. A sinusoidal compressive waveform was applied at 5% strain at 1 Hz for 20 cycles. Average stiffness was determined from the second through the final loading cycles.

### 2.8 | GAG quantification by DMMB assay

Dimethylmethylene blue (DMMB) assay was used following the culture period to measure the total GAG content in a subset of tested IVDs. IVDs were isolated, massed, and then digested in papain overnight at 65°C. The supernatant of samples was collected following centrifuging and plated alongside chondroitin sulfate standards (Sigma–Aldrich). Two hundred and fifty microliters of DMMB binding dye were added to each sample and samples were read at an absorbance of 525 nm using a spectrophotometer.

### 2.9 | Repeated hypo-osmolar exposure

FSUs underwent a 7-day preconditioning period in 400 mOsm media (representing approximately standard culture conditions) following extraction. FSUs were then placed in either a 400 mOsm or 200 mOsm (hypo-osmolar) media, with either DMSO (vehicle) or 10 μM GSK205, for 3 h each day for 5 consecutive days. Osmolarity was titrated by adding sucrose to the culture media.

### 2.10 | Sustained static loading of the functional spine units in culture

A custom platen-spring device was developed for loading the FSU and evaluating the role of TRPV4 in the load-mediated response of the IVD. The device utilized machined platens coupled with springs of known spring constants to deliver controlled loads based on the distance between the two platens. FSUs were statically loaded for 24 h at ~0.2 MPa, which is approximately two times the body weight of a mouse (~25 g) applied across the area of a lumbar IVD.^39^ DMSO (vehicle) or GSK205 (TRPV4 antagonist) were added to the medium of compressed IVDs during the entire duration of loading. Lipopolysaccharide (LPS) was added to a subset of IVDs for the positive controls for inflammation. Following the 24 h of loading, the device was removed and FSUs were cultured for 14 days. Following the culture period, samples were processed for histology, and media collected on Days 7 and 14 was measured for cytokine production.

### 2.11 | Histology and mouse IVD degeneration scoring

All samples were fixed in formalin and processed for paraffin embedding. Samples were then sectioned to 10 μm thickness and underwent Safranin O staining with a Fast Green counter stain. IVDs degeneration was quantified using a standardized scoring system.^40^ IVDs were graded by a single grader in a blinded fashion on three non-consecutive days. An intraclass correlation (ICC) was calculated between the scoring days to ensure consistent grading (Figure S1).

### 2.12 | Quantitative polarized light microscopy

Transmission-mode quantitative polarized light imaging (QPLI) was performed on Safranin-O stained sections of IVD as previously described.^41,42^ In brief, a SugarCube White LED Illuminator (Edmund Optics, Barrington, NJ, USA) with a Dolan-Jenner fiber optic backlight (Edmund Optics, Barrington, NJ, USA) was used to illuminate the histological section of interest. The light passed through a circular polarizing film (Edmund Optics, Barrington, NJ, USA) before being transmitted through the slide. The illuminated section was imaged using a division of focal plane polarization camera (FLIR, Wilsonville, OR, USA) with an achromatic 10x objective. The degree of linear polarization (DoLP) and the angle of polarization (AoP) were calculated on a pixel-wise basis for the entire field of view. DoLP corresponds to the strength of collagen fiber alignment and AoP corresponds to the orientation of collagen fibers. Images were manually segmented to select a region of interest spanning the AF. DoLP and AoP color maps were generated within the specified region of interest (ROI) and overlayed on the grayscale image for qualitative analysis. Average (AVG) DoLP and Standard deviation (STD) of DoLP values were calculated within the ROI.

### 2.13 | Statistical analysis

All statistical analysis was performed in either MATLAB or Graphpad Prism 9 software. Comparisons across experimental factors were conducted using student’s *t*-test or multi-way ANOVA as appropriate, and Tukey’s adjusted method was used for post hoc comparisons between groups. Results were considered statistically significant when *p* < .05.

## 3 | RESULTS

### 3.1 | TRPV4 activation induces Ca^2+^ signaling in IVD cells

**FIGURE 2:**
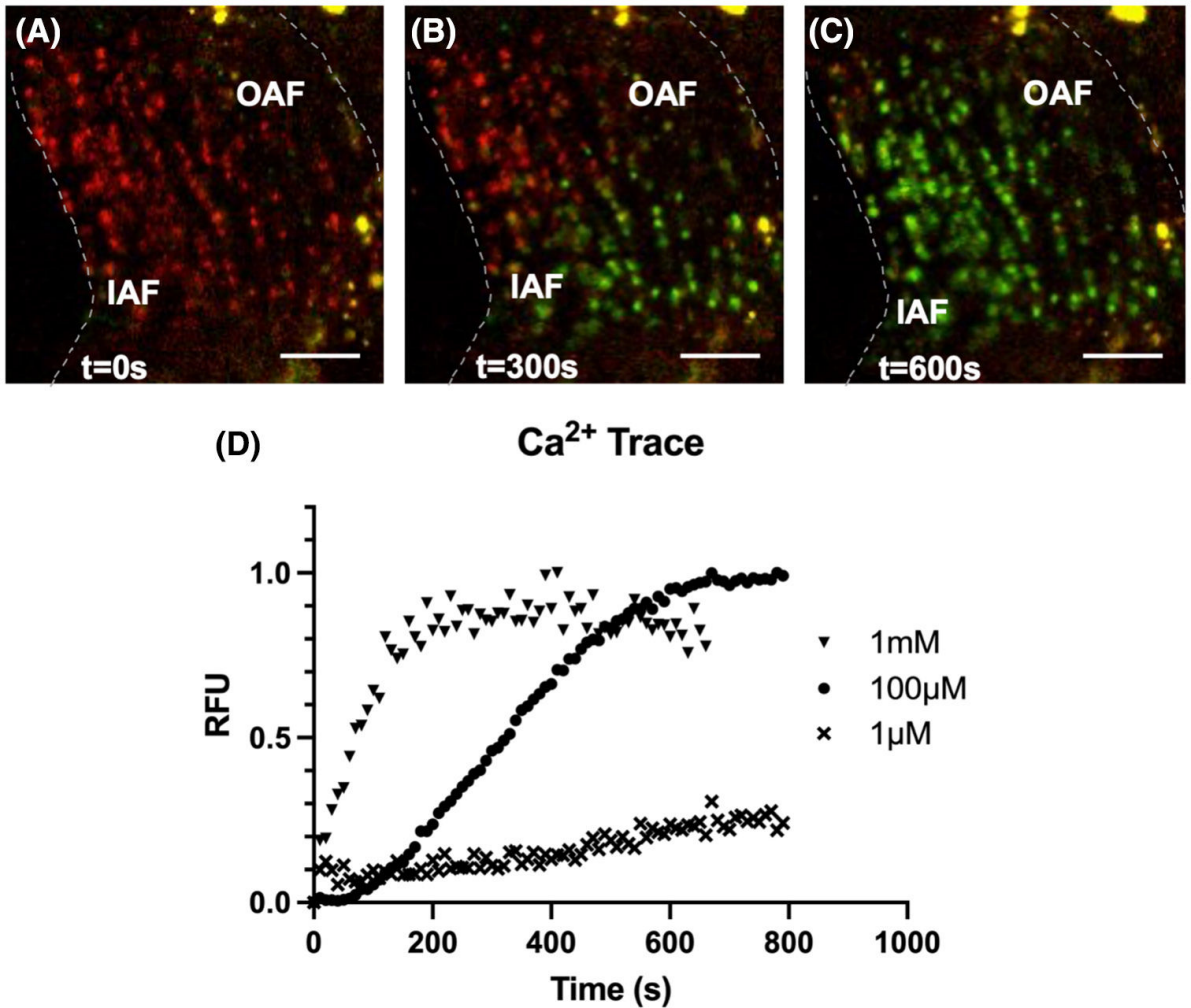
Representative confocal microscopy images showing that activation of TRPV4 by exposing the IVD to GSK101 rapidly increases intracellular Ca^2+^ ions. Images are from the sample treated with 100 μM of GSK101. The dotted lines indicate the borders of the annulus fibrosus, with IAF indicating the border of the inner annulus fibrosus, and OAF indicating the border of the outer annulus fibrosus. (A) The red signal indicates low intracellular Ca^2+^. (B) As the GSK101 gradually perfuses into the tissue to open TRPV4, the influx of intracellular calcium across the IVD cells is confirmed by the green fluorescence of the Fluo-4 Ca^2+^ indicator. (C) Within 600 s, the majority of cells show prevalent TRPV4 activation. (D) The time dependence and dose dependence of the resultant calcium flux are determined by the ratio between green and red fluorescence intensities at three different GSK101 concentrations. Scale bars are 100 μm.

To evaluate the role of TRPV4’s activation in Ca^2+^ signaling, the annulus fibrosis (AF) and nucleus pulposus (NP) cells in the intact IVD were observed with confocal microscopy using a ratio of intensities of fluorescent Ca^2+^ indicators. The addition of DMSO had no observable changes in fluorescence intensity (data not shown). The TRPV4 agonist GSK101 was added to the culture media, and then the fluorescent Ca^2+^ signal was first observed in the cells of the outer AF. The fluorescence then propagated radially inward toward the nucleus pulposus (Figure 2A–C; Video S1). The percentage of responsive cells increased in a dose-dependent manner. 1 μM of GSK101 elicited the slowest response that activated only 30% of cells (Figure 2D). More than 95% of observed cells experienced Ca^2+^ influx within 600 s of adding 100 μM GSK101 (Figure 2D). The 1 mM of GSK101 achieved greater than 95% activation in under 120 s. As GSK101 is a specific chemical activator of TRPV4, the observed intracellular Ca^2+^ flux confirms the presence and function of TRPV4 in both AF and NP cells. These Ca^2+^ fluxes typically resolved within an hour of TRPV4 agonist removal.

### 3.2 | NF-κB signaling is downstream of TRPV4 activation

**FIGURE 3:**
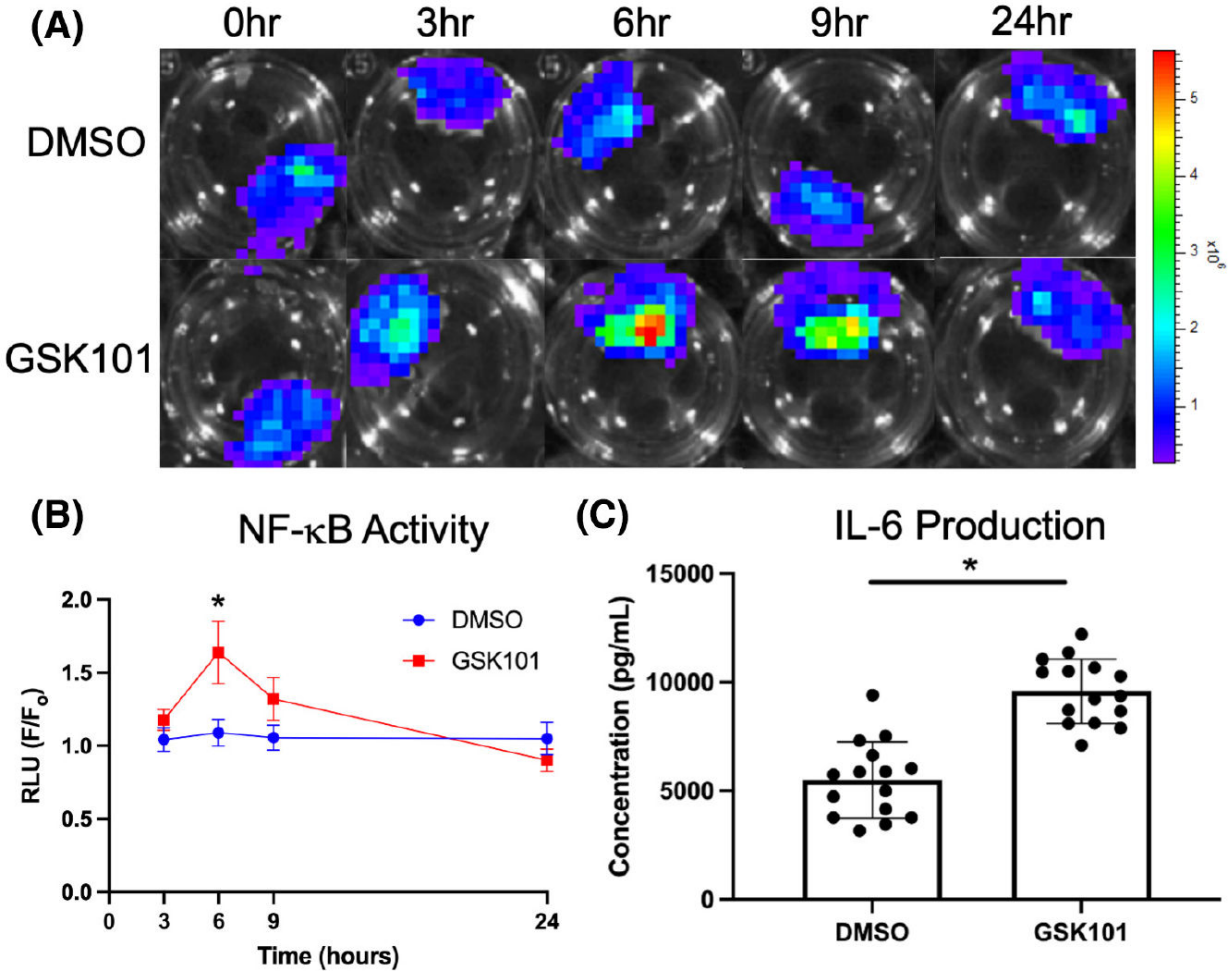
(A and B) NF-κB activity, reported by bioluminescence, increased significantly following GSK101 administration (*n* = 11) compared to DMSO (*n* = 10) at *t* = 6 h (*p* < .05), but this increase completely subsided by 24 h. (C) In a separate culture experiment (*n* = 15), administration of GSK101 to activate TRPV4 for 3 h each day for 7 days increased IL-6 production by the IVDs (*p* < .05). Data in (B) were statistically analyzed via one-way ANOVA with post hoc Tukey’s HSD where * indicates *p* < .05. Data in (C) were statistically analyzed via *t*-test where * indicates *p* < .05.

To evaluate the extent of NF-κB expression, the bioluminescence activity of the IVD was measured following a single bout of 3-h TRPV4 activation by GSK101. The monitoring of the subsequent 24-h time course revealed a transient peak at 6 h (*p* < .05) that gradually returned to baseline levels by 24 h post-GSK101 exposure (Figure 3A,B). The timing of the NF-κB peak signal typically lagged the peaks of the Ca^2+^ time course by an order of magnitude.

### 3.3 | Repeated TRPV4 activation elicits an inflammatory response and augments the ECM

**FIGURE 4:**
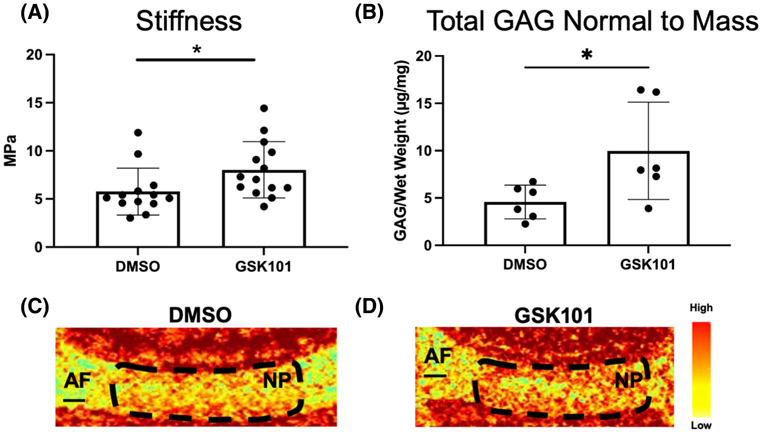
Administering the TRPV4 agonist GSK101 for 7 consecutive days for 3 h each day in the IVDs resulted in (A) an increase in average stiffness (*p* < .05) measured by dynamic compression and (B) an increase in total GAG content in the IVD (*p* < .05) measured by the DMMB assay. (C and D) Contrast-enhanced microCT (CEμCT) showed that TRPV4-agonized IVDs have high-attenuating nucleus pulposus indicating that there is increased hydration due to the higher GAG composition observed in (B). Data in (A) and (B) were statistically analyzed by ANOVA where * indicates *p* < .05. Scale bars in (C) and (D) are 200 μm.

Whereas a singular dose of GSK101 initiates a transient inflammatory response that is resolved rapidly, the repetitive activation of TRPV4 was applied to determine the effects of cytokine production. Seven days of repeated TRPV4 activation increased IL-6 production by the IVDs (Figure 3C) but not VEGFA (*p* = .41). Mechanical testing of these IVDs revealed increased compressive stiffness due to TRPV4 activation (Figure 4A). Those IVDs subjected to repeated TRPV4 activation exhibited significantly higher GAG content than the IVDs exposed to DMSO (Figure 4B), demonstrating that this repetitive TRPV4 activation promotes matrix deposition. CEμCT confirmed that the increase in GAG is primarily localized in the NP (Figure 4C,D).^43^

### 3.4 | TRPV4 inhibition has no dramatic effect on hypo-osmolarity-induced IL-6 production

**FIGURE 5:**
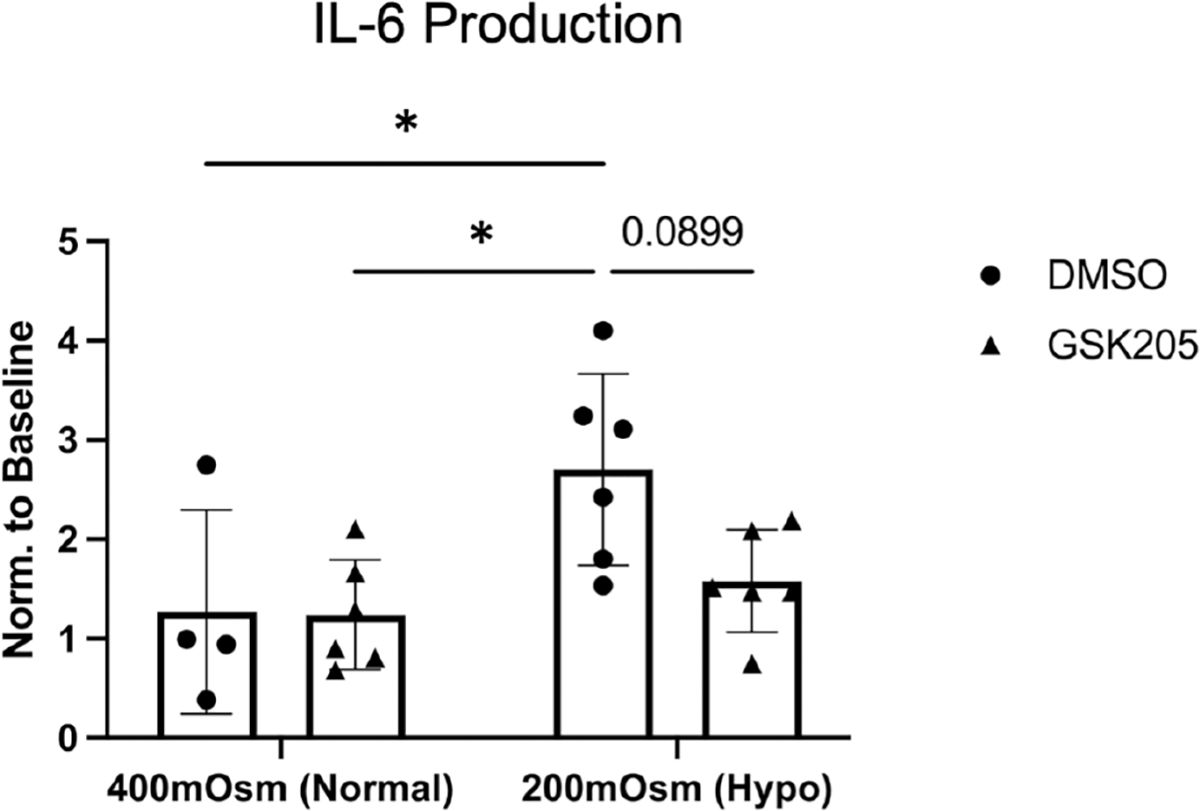
Exposing the IVD to hypo-osmolar pressures during culture significantly increased IL-6 production (*p* < .05). The elevated IL-6 production was not significantly resolved with the inhibition of TRPV4 by GSK205 (*p* = .09). Data were statistically analyzed via two-way ANOVA; post hoc comparisons were made by Tukey’s test.

FSUs were exposed to hypo-osmolar media (200 mOsm) for 5 consecutive days for 3 h each day, which significantly increased the production of IL-6 into the culture media at the end of the loading period (Figure 5). In those FSUs where the hypo-osmolarity was co-administered with the TRPV4 inhibitor, GSK205, the IL-6 cytokine concentration in the media was reduced by nearly 50% but not statistically significant (*p* = .09).

### 3.5 | Static loading-induced inflammation is partially resolved by TRPV4 inhibition

**FIGURE 6:**
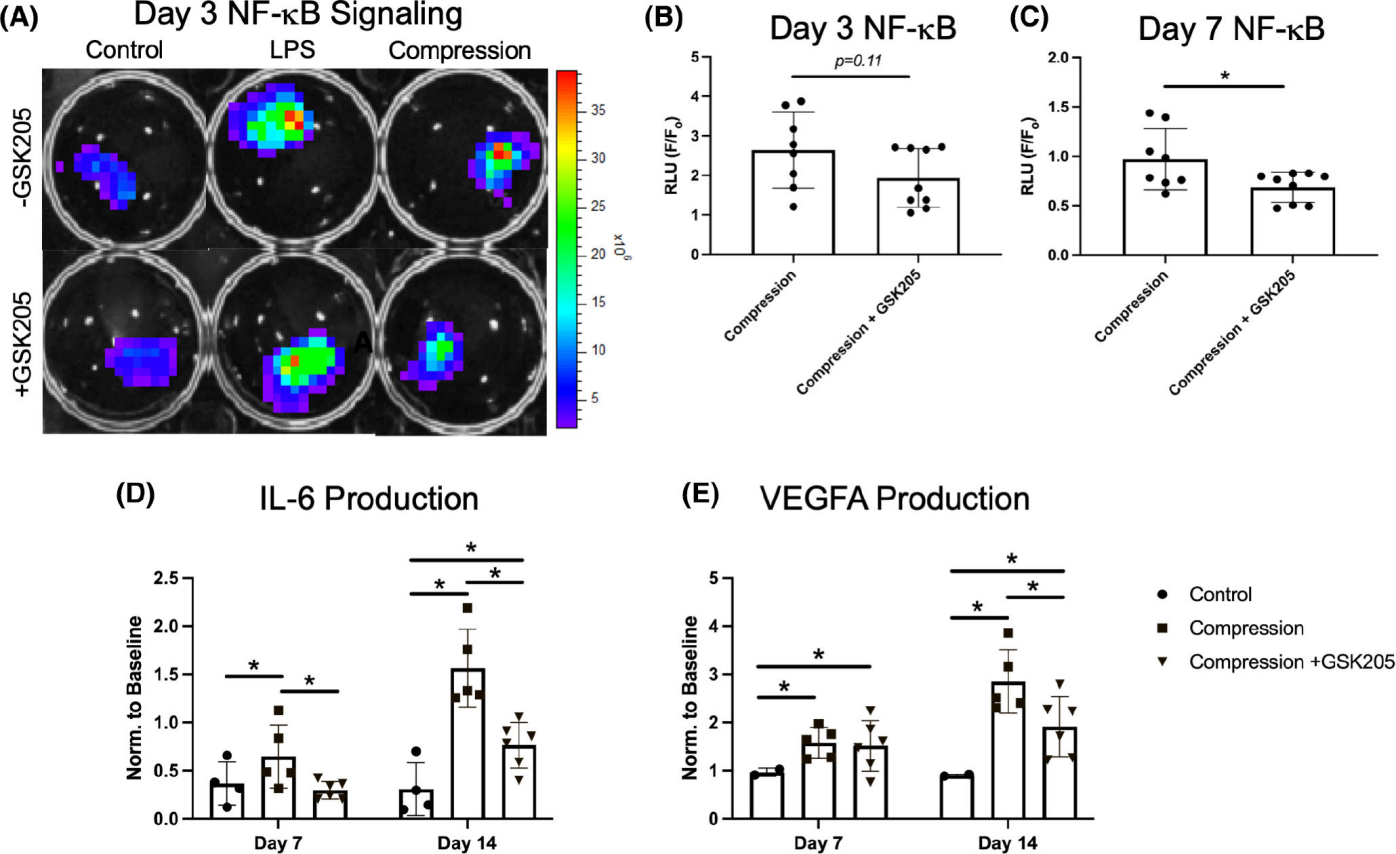
(A) Statically loaded IVDs (“Compression”) exhibited higher NF-κB activity than the unloaded control IVDs. Lipopolysaccharide was used as a positive control to induce the NF-κB luciferase activity. (B and C) NF-κB luciferase activity was reduced with inhibition TRPV4 by GSK205 (Day 3, *p* = .11; Day 7, *p* < .05). (D and E) The IVDs subjected to compression had increased levels of secreted IL-6 and VEGFA (*p* < .05) at both Day 7 and Day 14 of the culture. The increase was significantly reduced by TRPV4 inhibition on Day 7 for IL-6 and Day 14 for both IL-6 and VEGFA (*p* < .05). Values were normalized to Day 0 media. Data in (B) and (C) were statistically analyzed via unpaired *t*-test where * indicates *p* < .05. Data in (D) and (E) were statistically analyzed via one-w ay ANOVA with post hoc Tukey’s HSD where * indicates *p* < .05.

To evaluate the role of TRPV4 in the IVD load-mediated response, FSUs were observed for 14 days following a 24-h static loading regimen. The loaded FSUs exhibited a significant increase of NF-κB signaling at day 3 of the organ culture compared to the non-loaded control (Figure 6A), through the inhibition of TRPV4 only modestly suppress this NF-κB elevation (Figure 6B). However, by day 7, NF-κB signaling was blunted with TRPV4 inhibition (Figure 6C). Further, concentrations of IL-6 and VEGF-A were both significantly increased at Days 7 and 14 of culture in the loaded FSUs as compared to the control (Figure 6C). Notably, IL-6 concentration was significantly lower with TRPV4 inhibition on both Days 7 and 14. The inhibition of TRPV4 had no effect on VEGFA concentration on Day 7, however, it significantly reduced VEGFA on Day 14 compared to the loading condition without GSK205.

**FIGURE 7:**
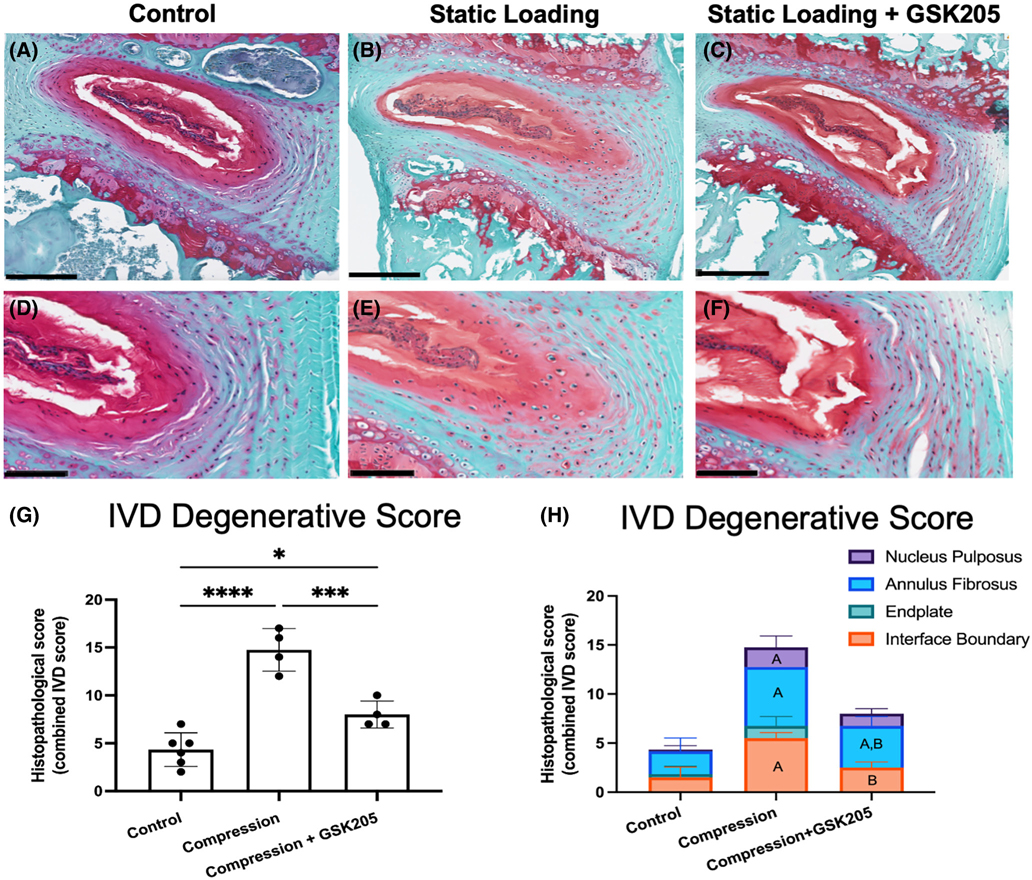
(A–C) The Safranin-O stained histological sections revealed degenerative changes in the compressed IVDs (14 days of loading) that were absent in the non-loaded IVDs. Compressed IVDs with TRPV4 inhibition were partially protected from these degenerative changes. Scale bars are 200 μm. (D–F) Qualitatively, the most apparent changes in the loaded IVDs were the cell morphology of inner AF cells as well as the boundary between the AF and NP. (G) The degenerative changes in the loaded IVDs were significantly higher than both the controls and the TRPV4 inhibition groups. (H) The protection against degeneration in the TRPV4-inhibited IVDs was primarily in the endplate as well as the boundary interfaces of the IVD (A indicates a difference with the control group; B indicates differences with the compression group). Data in (G and H) were statistically analyzed via ANOVAs respectively with post hoc Tukey’s HSD where * indicates *p* < .05. Scale bars are 100 μm.

Histological evaluation of compressed IVD revealed a deterioration of the NP-AF boundary, rounded AF cells, and moderate fibrosis in the NP (Figure 7A–F). TRPV4 inhibition by GSK205 in compressed IVDs maintained the distinct boundary between the NP and AF and reduced the number of rounded AF cells (a hallmark of degeneration) (Figure 7B,C,E,F). The degeneration of the IVDs was quantified using a standardized scoring system for mouse IVDs.^40^ IVDs that underwent compression exhibited moderate-to-high degeneration that was significantly higher than control IVDs (Figure 7G). The compressed IVDs with TRPV4 inhibition exhibited mild degeneration. Despite these degenerative characteristics in comparison to the control IVDs, the degeneration score was significantly lower than the compressed IVDs with intact TRPV4 function. Using the histologic scoring system, the individual components of the IVD were assessed (Figure 7H). The IVDs with TRPV4 inhibition had lower degenerative scores for the end plates, NP-AF boundary, and AF. However, there was little change in the NP degenerative score between the two compressed groups.

### 3.6 | TRPV4 inhibition does not protect against loading-induced collagen disorganization

**FIGURE 8:**
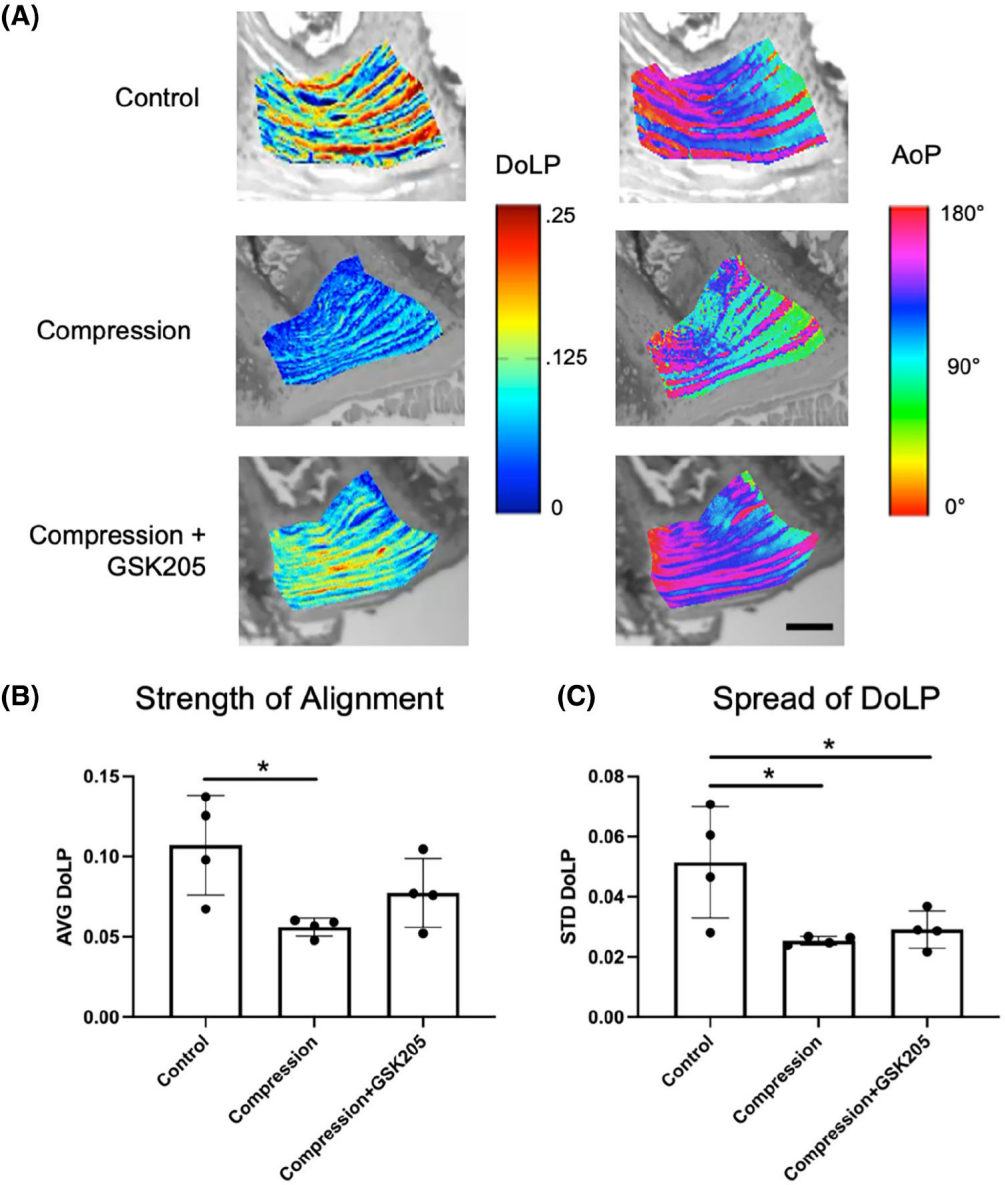
(A) Representative color maps from quantitative polarized light imaging (QPLI)showing the degree of linear polarization (DoLP) and the angle of polarization (AoP) for control, compression, and compression + GSK205 groups. The DoLP represents the strength of collagen fiber alignment, and the AoP is indicative of fiber orientation. The scale bar is 200 μm. (B) Average (AVG) DoLP, showing the average strength of alignment, was decreased within the AF-spanning ROI in the compression group compared to controls. When TRPV4 was inhibited via GSK205, there was no observable difference in AVG DoLP compared to controls. (C) Standard deviation (STD) DoLP, representing the relative spread in DoLP values, was decreased in both experimental groups compared to controls. Data in (B) and (C) were statistically analyzed via one-way ANOVA with post hoc Tukey’s HSD where * indicates *p* < .05.

Quantitative polarized light imaging (QPLI) was used to evaluate the organization of collagen fibers in the AF through the measurements of degree of linear polarization (DoLP) and the angle of polarization (AoP) (Figures S3 and S4). DoLP measures the strength of the alignment of collagen fibers and is related to the local retardance and thus structural anisotropy of the tissue: a DoLP of 1 indicates a uniformly aligned tissue whereas a DoLP of 0 indicates isotropic collagen organization. AoP values provide a measurement of the orientation of collagen fibers. Healthy AF can be characterized by a highly organized, lamellae structure of collagen fibers. Inspection of the AoP shows alternating layers of collagen fiber directionality, indicating the primary collagen fiber directions of the alternating lamellae. There were no observed qualitative changes in the AoP or fiber orientation between groups as each showed distinct layers of alternating alignment of approximately equal thickness and spacing (Figure 8A). The compressed IVDs had significantly (*p* < .05) decreased average DoLP (AVG DoLP) compared to uncompressed controls, indicating a less strongly aligned tissue (Figure 8B). Compressed IVDs with TRPV4 inhibited did not have significantly decreased average DoLP. In addition, compression decreased the standard deviation of the DoLP (STD DoLP) regardless of TRPV4 inhibition (Figure 8C), indicating decreased uniformity of the collagen fiber alignment in the IVD. Similar levels of STD DoLP for both compression groups indicate similar lamellar tissue structure with or without TRPV4 inhibition. However, inhibiting TRPV4 in compressed IVDs did not rescue collagen disorganization, suggesting that the effects on collagen architecture may be a direct mechanical consequence of loading rather than due to TRPV4-mediated responses.

## 4 | DISCUSSION

Our results indicate TRPV4 plays a role in mechanotransduction in response to physiologically relevant loading of the IVD. The effects of TRPV4 on the IVDs depended on the repetition, concentration, and loading history. TRPV4 activation by the small molecule agonist GSK101 in native IVD tissue rapidly initiated Ca^2+^ signaling in AF cells in a dose-dependent manner. NF-κB activity and subsequent IL-6 production also increased following transient activation of TRPV4. The repeated activation of TRPV4 had positive effects on the ECM of the IVD, including improved whole-IVD compressive stiffness and increased glycosaminoglycans in the NP. The prolonged daily repeated exposure to GSKS101 sustained the increase in TRPV4-related outcomes.^27^ Additionally, exposing the IVD to repetitive hypo-osmolar conditions resulted in IL-6 production similar to a single bout of TRPV4 activation. Suppression of TRPV4 concomitant with the hypo-osmolar exposure of the IVDs reduced the IL-6 synthesis.

The sustained physiologic compression of the whole IVD recapitulated some aspects of TRPV4 activation, including NF-κB signaling and IL-6 production. However, sustained compression also created hallmarks of degeneration and microstructural disorganization.^40^ Applying a TRPV4 antagonist during sustained compression partially alleviated the degeneration and the matrix disorganization. Production of cytokines such as VEGFA likely exacerbated any mechanical damage that occurred within the IVD. There were also notable regional variations in degeneration. In particular, TRPV4 inhibition appeared more effective in regions of higher endogenous expression of TRPV4,^34^ with the greatest protective effect in the inner AF. Yet TRPV4 inhibition only conferred partial protection from the degeneration in the outer AF and the interface boundaries. Qualitatively, polarized light imaging revealed greater disorganization in the AF in compressed IVDs.^41^ TRPV4 inhibition did not improve collagen alignment of the compressed IVDs. Along with histopathology scoring, the results suggest that the timely inhibition of TRPV4 can dampen the inflammatory effects on degeneration but not load-mediated mechanical damage. It is worth noting that the inhibition of TRPV4 for 7 days depressed NF-κB signaling beyond the baseline, and since NF-kB has critical roles in cell survival, the long-term suppression of NF-kB via inhibiting TRPV4 should be applied cautiously.

Similar to articular chondrocytes, activation of TRPV4 in the IVD promoted robust NF-κB signaling that subsided in 24 h,^44,45^ which is a downstream target of CaMKII and calcium signaling following TRPV4 activation.^46–49^ Though the precise role of NF-κB is not known here, NF-κB is canonically required to initiate a regenerative response during tissue repair.^50–52^ Consistent with these observations, we observed a spike in NF-κB activity with TRPV4 activation and sustained mechanical loading, followed by improvements in mechanical function and greater GAG density in the NP. Similar to NF-κB, IL-6 can invoke reparative responses in multiple tissues,^53–55^ and it has pleiotropic effects on a myriad of cell types including immune cells and neurons. Similar to our observations, IL-6 has been reported to promote GAG synthesis in fibroblasts.^56^ Conversely, the persistent elevation of IL-6 and other inflammatory factors is linked to chronic painful intervertebral disc diseases and promotes ECM catabolism.^57–59^ Our data here suggest that the TRPV4-dependent activation of IL-6 results in a net increase in GAGs between these known anabolic and catabolic functions. Hypo-osmotic conditions in IVD cells have also stimulated IL-6 expression independently of the TRPV4-pathway, indicating multiple compensatory mechanisms can activate IL-6.^60^

TRPV4 is a critical osmotic and mechanical regulator in multiple organ systems and cell types,^24–26^ and we demonstrate here that it robustly responds to mechanical loading in the intact IVD. There are also a number of known mechanoreceptors, including the TRP family and PIEZO ion channels that regulate Ca^2+^ flux, that could participate in IVD’s adaptation to sustained loading.^61,62^ In particular, the small molecule inhibitor GSK205 also cross-reacts with TRPA1, inhibiting the ion channel, and other ion channels may have contributed to the observed effects reported in this study.^63^ However, it is worth noting that TRPA1 is primarily expressed in sensory neurons and not in healthy mature IVDs, and therefore is unlikely to be present in the IVDs or influence our ex vivo cultures.^64,65^ Moreover, our organ approach here allowed us to selectively isolate and control the IVD cells in their native ECM, ensuring the coupling of our observations between sustained mechanical loading, TRPV4 inhibition, and cellular responses on the IVD tissue. Our results here suggest that TRPV4 is a crucial target for the mechano-responsiveness of the IVD and that modulation of TRPV4 may provide a potential therapeutic target against deleterious loading.

## Supplementary Material

Figure S1

Video S1

## Data Availability

The data that support the findings of this study are available in the methods and/or supplementary material of this article.

## Abbreviations:

AF: annulus fibrosus
AoP: angle of polarization
Ca: calcium ion
CEμCT: contrast-enhance micro-computed tomography
DMMB: dimethylmethylene blue
DoLP: degree of linear polarization
ECM: extracellular matrix
EP: endplate
FSU: functional spine unit
GAG: glycosaminoglycan
ICC: intraclass correlation
IL: interleukin
IVD: intervertebral disc
LBP: low back pain
LPS: lipopolysaccharide
NF-κB: nuclear factor κB
NP: nucleus pulposus
QPLI: quantitative polarized light imaging
ROI: region of interest
TRPV4: transient receptor potential vanilloid 4
VEGF: vascular endothelial growth factor
